# Work Hardening Behavior and Microstructure Evolution of a Cu-Ti-Cr-Mg Alloy during Room Temperature and Cryogenic Rolling

**DOI:** 10.3390/ma16010424

**Published:** 2023-01-02

**Authors:** Rong Li, Zhu Xiao, Zhou Li, Xiangpeng Meng, Xu Wang

**Affiliations:** 1 School of Materials Science and Engineering, Central South University, Changsha 410083, China; 2 Key Laboratory of Non-Ferrous Metal Materials Science and Engineering, Ministry of Education, Changsha 410083, China; 3 State Key Laboratory for Powder Metallurgy, Central South University, Changsha 410083, China; 4 Ningbo Boway Alloy Material Co., Ltd., Ningbo 315135, China

**Keywords:** Cu-Ti-Cr-Mg alloy, cryogenic rolling, hardness, texture, twins

## Abstract

A Cu-1.79Ti-0.39Cr-0.1Mg (wt.%) alloy was prepared by a vacuum induction melting furnace in a high-purity argon atmosphere. The effects of room temperature rolling and cryogenic rolling on the microstructure, textures, and mechanical properties of the alloy were investigated by means of electron backscatter diffraction, transmission electron microscopy, and X-ray diffraction. The results show that the hardness of the cryogenically rolled alloy is 18–30 HV higher than that of the room temperature rolled alloy at any tested rolling reduction. The yield strength and tensile strength of the alloy cryogenically rolled by 90% reduction are 723 MPa and 796 MPa, respectively. With the increase of rolling reduction, the orientation density of the Cube texture decreases, while the Brass texture increases. The Brass texture is preferred especially during the cryogenic rolling, suggesting that the cross-slip is inhibited at the cryogenic temperature. The dislocation densities of Cu-Ti-Cr-Mg alloy increase significantly during the deformation, finally reaching 23.03 × 10^−14^ m^−2^ and 29.98 × 10^−14^ m^−2^ after a 90% reduction for the room temperature rolled and cryogenically rolled alloys, respectively. This difference could be attributed to the impediment effect of cryogenic temperature on dynamic recovery and dynamic recrystallization. The cryogenic temperature promotes the formation of the dislocation and the nano-twins, leading to the improvement of the mechanical properties of the alloy.

## 1. Introduction

Elastic copper alloys with ultra-high strength have been widely used in electronics, aerospace, and marine engineering fields [1,2,3]. Copper beryllium alloy is the typical elastic alloy with a good combination of strength and conductivity, however, the high carcinogenicity of the Be limits the application of Cu-Be alloy in environment-friendly areas. Cu-Ti alloy possesses ultra-high strength, good elasticity, and moderate electrical conductivity, and has been an ideal candidate for the replacement of Cu-Be alloy [4,5].

In recent years, much work has been made to further improve the comprehensive properties of Cu-Ti alloy through either composition design or thermo-mechanic treatment. Typically, a reduction of Ti content in Cu-Ti alloy could intensively increase the conductivity of the alloy, however, this could also reduce the strength of Cu-Ti alloy [6]. Markandeya et al. reported that the addition of the Cr element could improve the yield strength and tensile strength of Cu-Ti alloy [7]. Li et al. investigated the microstructure and properties of Cu-2.7Ti-0.15Mg-0.1Ce-0.1Zr alloy and found that the addition of trace Mg element could improve the strength and conductivity of Cu-Ti alloy due to the dragging effect of Mg atoms [8]. Rouxel et al. found that the addition of trace Fe element in Cu-Ti alloy inhibited the modulation decomposition at the early stage of aging, leading to improvements in ductility and strength [9].

For metallic materials, work hardening is a common method to improve the microstructure and mechanical properties of the alloys [10,11]. Severe plastic deformation has been proven to be an ideal method to obtain the sub-micron or nanocrystalline grain structures, leading to the improvements of the room temperature strength and high-temperature ductility of copper alloys [12]. So far, several plastic deformation technologies have been developed to improve the mechanical properties of the alloys. The typical SPD methods include equal-channel angular pressing [13], groove rolling [14], accumulative roll-bonding [15], high-pressure torsion [16], slope extrusion [17], and cryogenic rolling. In most cases, it is difficult to fabricate large-sized products with uniform microstructure by equal-channel angular pressing, slope extrusion, and high-pressure torsion, and the materials prepared by accumulative roll-bonding often have low inter-facial bonding strength and poor edge quality. In recent years, cryogenic temperature rolling (CTR) has been attracted much attention and considered a promising industrial production method to produce alloy [18,19]. Cryogenic temperature rolling can inhibit dynamic recovery and dynamic recrystallization, and effectively increase the dislocation density [20], promoting the formation of an ultra-fine grain of the alloys. Especially, cryogenic temperature rolling could also promote the formation of deformation twins and stacking faults into the matrix [21], and the increased resistivity of the twin boundaries could be about an order of magnitude lower than that from the grain boundaries [22], improving the comprehensive properties of metallic materials. Shanmugasundaram et al. investigated the effect of cryorolling, low-temperature annealing, and aging treatments on the microstructure and mechanical properties of Al-Cu alloy, and the ultra-fine crystal structure and excellent properties were obtained through the cryogenic temperature rolling [23]. Yu et al. found that the asymmetric-cryorolled sheet had higher strength and better thermal stability compared to the asymmetric-rolled sheet [24]. Wang et al. prepared Cu-35Zn alloy with a good combination of strength and ductility due to the ultra-fine microstructure, high-density dislocations, and nanometer-scale deformation twins [25]. However, there was little work on the investigation of the microstructure evolution of Cu-Ti alloy during cryogenic rolling.

The typical Cu-Ti-based alloys included Cu-1.8Ti and Cu-2.4Ti alloys. In this paper, Cu-1.8Ti alloy was chosen due to its relatively low Ti content and high electrical conductivity. The addition of the Cr element could improve the comprehensive performance of Cu-Ti alloy, and the content of Cr was determined to be 0.4 wt.% according to its solid-solution limitation in the copper matrix. It had been widely reported that the addition of micro-alloying Mg element had a dragging effect on dislocation in copper alloys. As a typical trace element, its content was often chosen between 0.05–0.15 wt.% Mg, and we chose 0.1 wt.% in this study. Due to the low Ti element content, the strength of the alloy prepared by room temperature rolling could sometimes not meet the performance requirements in some specific application scenarios. Therefore, cryogenic rolling was used in this study in order to further improve the strength of the alloy. The evolution of microstructure, textures, and properties of the alloy during rolling was investigated in details, which could be expected to provide a theoretical basis for improving the comprehensive performance of Cu-Ti-based alloys.

## 2. Materials and Experimental Procedures

The Cu-Ti-Cr-Mg alloy was prepared by vacuum induction melting furnace in a high-purity argon atmosphere to avoid oxidation, with the raw materials of pure copper (99.9%), pure titanium (99.9%), pure chromium (99.9%) and Cu-20 wt.% Mg master alloy, and all the chemical components were placed in the crucible before melting. The measured compositions were detected by inductively coupled plasma-optical emission spectroscope (ICP-OES). The nominal and measured compositions were shown in Table 1. After removing the defects on the surface, the ingot was homogenization-treated at 880 °C for 6 h, and then hot-rolled with a reduction of 60%. The hot-rolled samples should be solid-solution treated to ensure that the solute atoms were completely dissolved into the matrix, and the hot deformation microstructure could be completely recrystallized. The solid-solution temperature and soaking time were 880 °C and 2h, respectively. Then the samples were rolled at room temperature and cryogenic temperature, respectively. In order to investigate the microstructure evolution of the alloy during rolling, the solid-solution treated samples were rolled with different reductions of 30%, 60%, 80%, and 90%, respectively. A handheld infrared thermometer was used to measure the temperature value of the alloy before each pass of rolling, keeping the cryogenic temperature at about −50 °C. After each pass of cryogenic rolling, the rolled samples were immediately immersed in liquid nitrogen and kept for at least 10 min to warrant the temperature of the samples at a low level. The total processing time of each cryogenic rolling pass (refers to the time when the samples were exposed to the room temperature environment during rolling) was less than 30 s. All rolling processes were carried out on a four-high rolling mill with Φ80 − Φ120 × 480 mm/Φ300 − Φ350 × 450 mm. For simplification, the room temperature rolled samples and cryogenic temperature rolled samples are abbreviated to RTR samples and CTR samples, respectively. For example, the sample with cryogenic rolling by 30% reduction is abbreviated to CTR30, as shown in Table 2.

**Figure 1:**
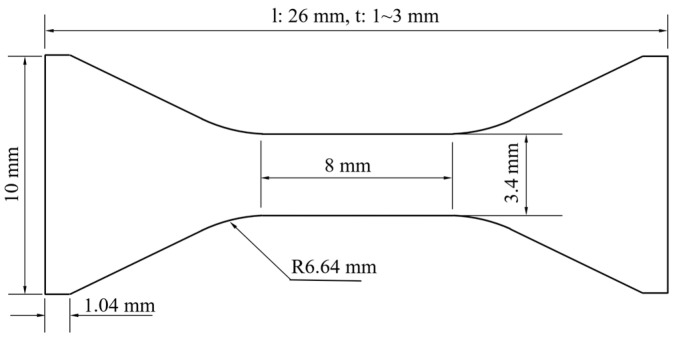
The dimensions of mechanical property test samples.

The texture measurement was carried out on the D8 Discover X-ray diffractometer, with a sample size of 12 mm (RD) × 10 mm (TD). The orientation distribution function (ODF) plots ($\varphi_{2}$= 0°, 45° and 60°) were calculated by the {200}, {220}, {111} pole diagrams according to the Bundle method. The hardness measurement was conducted on the HV-1000 microhardness tester, with a load of 1 kg and a dwell time of 15 s. Each sample was measured at least five times, and the average value was taken. The mechanical performance test was carried out at room temperature using an MTS-810 mechanical property tester following the GB/T 228.1-2010, with a strain rate of 0.5 min^−1^. The dimensions of the samples tested were shown in Figure 1.

The microstructure in the longitudinal section was observed using Leica EC3 optical microscopy, FEI Helios Nanolab 600i scanning electron microscope with a NordlysMax^2^ electron backscatter diffraction (EBSD) detector, and FEI Tecnai G^2^ F20 field-emission transmission electron microscope (TEM).

Metallographic samples were prepared by mechanical polishing and then corroded in an aqueous solution of ferric chloride and hydrochloric acid (FeCl_3_:HCl:CH_3_CH_2_OH = 5 g:25 mL:100 mL). EBSD was observed on the RD-ND plane (RD is the rolling direction, ND is the normal direction, and TD is the transverse direction of the samples). EBSD samples were prepared by mechanical polishing, and then electropolishing in an aqueous phosphoric acid solution of 70% volume fraction with a voltage of 2 V and a polishing time of 30 s. TEM samples were prepared by ion bean thinning.

## 3. Results

### 3.1. Microstructure of the Casted, Homogenization, Hot-Rolled, and Solid-Solution Treated Alloys

**Figure 2:**
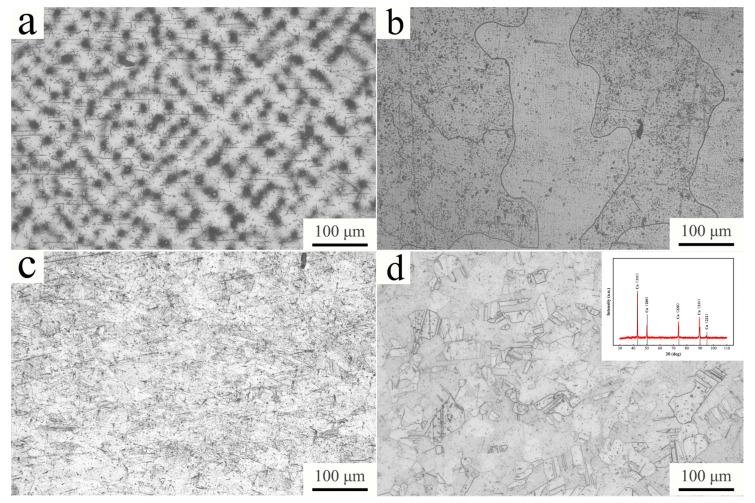
Metallograph of (**a**) casted, (**b**) homogenization, (**c**) hot-rolled, and (**d**) solid-solution-treated Cu-Ti-Cr-Mg alloy.

Figure 2a shows the typical metallograph of the casted Cu-Ti-Cr-Mg alloy. The sample shows the dendritic structure with a discontinuous pit-like characteristic, which resulted from the segregation of titanium. Normally, the electrode potential of titanium is lower than that of copper, so it is often corroded as an anode. Therefore, the dendritic structure under the metallograph is pitted. Figure 2b shows the metallograph of the sample after homogenization treatment, and the dendrite segregation is eliminated in the sample. After hot-rolled, the grain size of the sample is greatly reduced, while a partially deformed microstructure is observed (shown in Figure 2c). The metallograph of the solid-solution treated sample is shown in Figure 2d, presenting a homogeneous structure with a large number of annealed twins. The deformation microstructure after hot rolling treatment is basically eliminated. The inserted image on the right corner of Figure 2d shows the XRD diffraction pattern of the solid-solution treated sample. Only the characteristic diffraction peaks of copper appear in the inserted image in Figure 2d, indicating that the solid-solution treated sample is a single-phase solid-solution of copper.

### 3.2. Properties of Deformed Alloy

**Figure 3:**
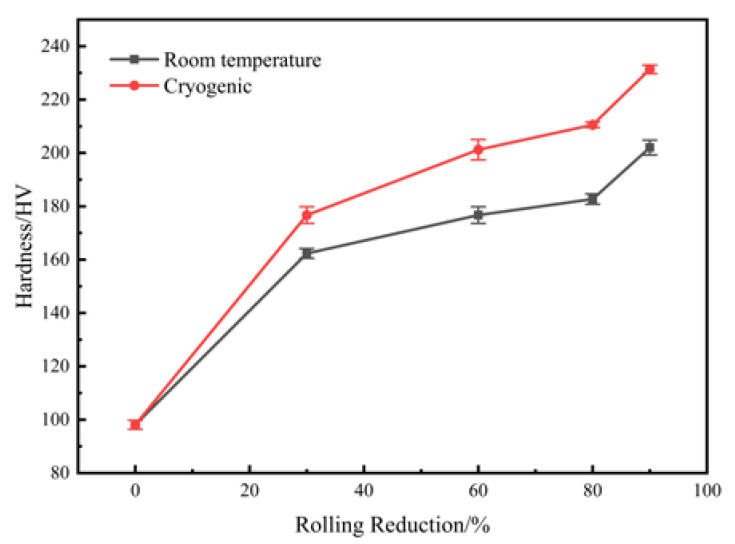
The variations of hardness with different rolling reductions.

Figure 3 shows the variations of hardness with different rolling reductions at room temperature and cryogenic temperature. The average hardness of the solid-solution treated sample is 98 HV. After a rolling reduction of 30%, the hardness of the samples increases significantly. With the further increase of rolling reduction, the rising trend of hardness begins to slow down, but it still shows an upward trend. At any given rolling reduction, the hardness of CTR samples is always larger than that of RTR samples. The results show that the hardness of the cryogenically rolled alloy is 18–30 HV higher than that of the room-temperature rolled alloy at any tested rolling reduction. Compared with room temperature rolling, Cu-Ti-Cr-Mg alloy has a higher work hardening rate at cryogenic temperature. The average hardness of the CTR90 sample is up to 231 HV, which is about 29 HV higher than that of the RTR90 sample.

The yield strength, tensile strength, and elongation of the solid-solution treated sample, RTR samples, and CTR samples with different rolling reductions are shown in Table 3. The solid-solution treated sample has a relatively low tensile strength of 319 ± 30 MPa but a high elongation of 46.8 ± 0.3%. The strength of both RTR and CTR samples increases significantly with the increase of rolling reduction, but the increase in strength is accompanied by a decrease in the ductility of the alloy. Compared with RTR samples, CTR samples have higher strength at any given rolling reduction. However, the elongation of CTR samples is close to that of RTR samples. The yield strength and tensile strength of the CTR90 sample are 723 MPa and 796 MPa, increasing by about 25% and 23% compared with those of the RTR90 sample, respectively. This suggests that CTR treatment can improve the mechanical properties of Cu-Ti-Cr-Mg alloy more effectively than RTR treatment. It is worth noting that the elongations of both the RTR samples and CTR samples reach a minimum value at a rolling reduction of 80%.

### 3.3. Evolution of Microstructure during the Rolling Process

#### 3.3.1. Metallograph Observation

**Figure 4:**
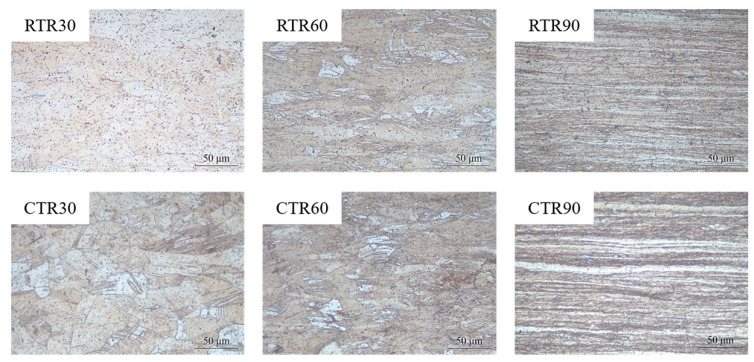
Metallograph of Cu-Ti-Cr-Mg alloy with different rolling conditions.

Figure 4 shows the metallograph of Cu-Ti-Cr-Mg alloy with different rolling treatments. In the RTR process, very few deformation structures appear in the alloy with a rolling reduction of 30%. When the rolling reduction increases to 60%, the grains are elongated along the rolling direction, and deformation twins appear in some grains. Further increasing rolling reduction to 90%, the grains become much thinner, and the fibrous structure forms. The CTR process has a similar microstructure evolution to the RTR process. However, it is noted that a large number of deformation twins appear in the CTR30 sample.

#### 3.3.2. XRD and EBSD Analysis

**Figure 5:**
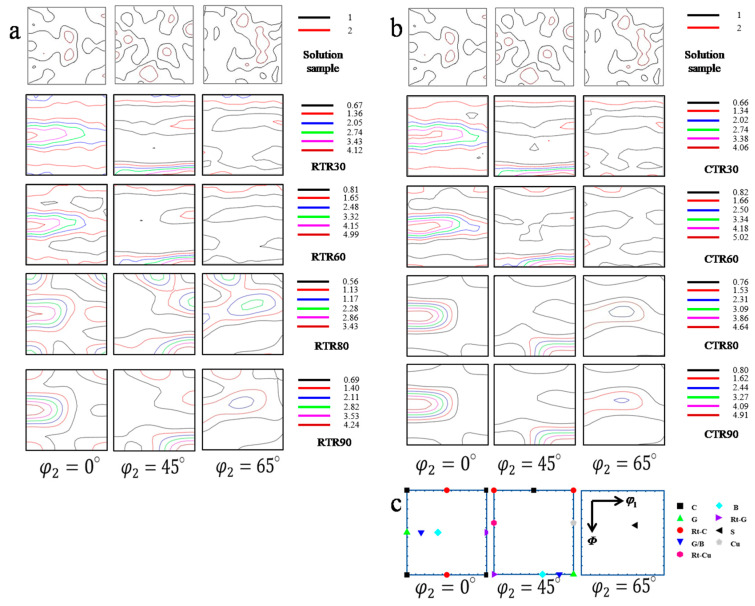
ODF plots of rolled samples at (**a**) room temperature and (**b**) cryogenic temperature; (**c**) schematic of the position of different texture components on the ODF section (φ_2_ = 0°, φ_2_ = 45°, and φ_2_ = 65°).

Figure 5 shows the typical texture composition of the face-centered cubic metal during the rolling process and the orientation distribution function of the Cu-Ti-Cr-Mg alloy rolled at room temperature and cryogenic temperature (φ_2_ = 0°, 45°, and 65°), respectively. The results show that the rolling reduction and rolling temperature have obvious effects on the texture evolution of the rolled samples. The orientation density of the solid-solution treated samples (0% reduction) is low, and there are a small number of Cube texture, weak Brass texture, and weak S texture. Prominent Brass texture, Goss texture, and a small number of S texture begin to appear in RTR30 and CTR30 samples. With the increase of rolling reduction, the orientation density of Brass texture, Goss texture, and S texture in the samples increases, while that of Cube texture decreases. The Cube texture is barely visible in the CTR90 sample. At any given rolling reduction, the orientation density of several major textures in the CTR samples is higher than that in the RTR samples.

EBSD analysis was used to compare the grain size and twin content of the samples rolled at different temperatures (represented by samples with a reduction of 60%).

**Figure 6:**
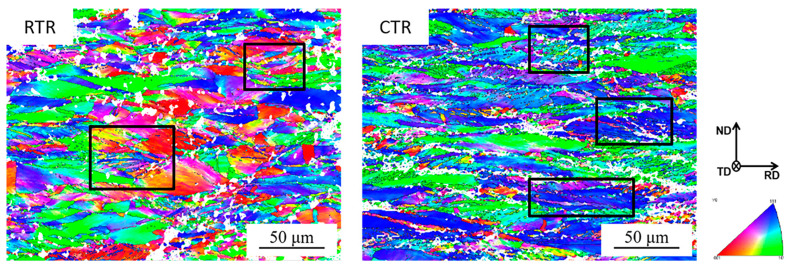
IPF of RTR and CTR Cu-Ti-Cr-Mg alloy with a rolling reduction of 60%.

Figure 6 shows the inverse pole figure (IPF) map of RTR and CTR samples with a rolling reduction of 60%. It is evident that grains are elongated along the rolling direction in both samples. The grains of the sample deformed at cryogenic temperature have a greater aspect ratio, indicating that the deformation of the sample at cryogenic temperature is much more serious. Moreover, there is a higher density of twins in the CTR sample (as shown in the box in Figure 6).

**Figure 7:**
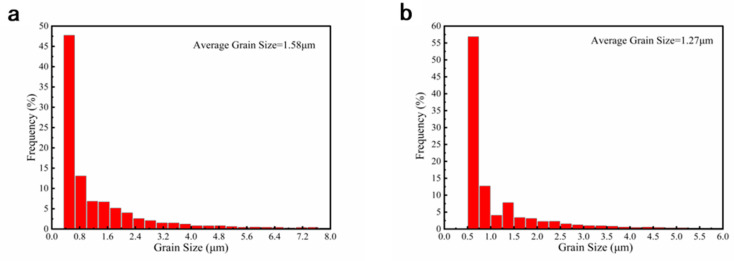
Grain sizes distribution of (**a**) RTR and (**b**) CTR Cu-Ti-Cr-Mg alloy with a rolling reduction of 60%.

The distribution of grain sizes of RTR and CTR samples with a rolling reduction of 60% is shown in Figure 7. After 60% deformation, the grains in both samples are broken into small sizes. The fraction of grains of the CTR sample with the sizes of 0.5 μm is up to 55%, higher than that of the RTR sample. The average grain sizes of RTR and CTR samples are 1.58 μm and 1.27 μm, respectively.

**Figure 8:**
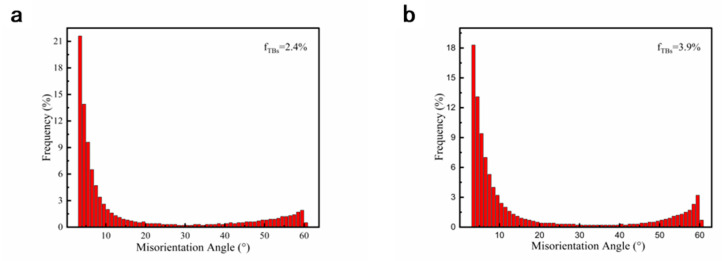
Distribution of boundary misorientation angle of (**a**) RTR and (**b**) CTR Cu-Ti-Cr-Mg alloy with a rolling reduction of 60%.

Figure 8 shows the distribution of boundary misorientation angle of RTR and CTR samples with a rolling reduction of 60%. Two peaks are shown at a low angle and a high angle of 60°, respectively, corresponding to the broken grains with small misorientation and the deformation twins. Comparing the twin densities of RTR and CTR samples, the percentage of twin boundaries in the CTR sample (3.9%) is slightly higher than that of the RTR sample (2.4%).

#### 3.3.3. TEM Observation

**Figure 9:**
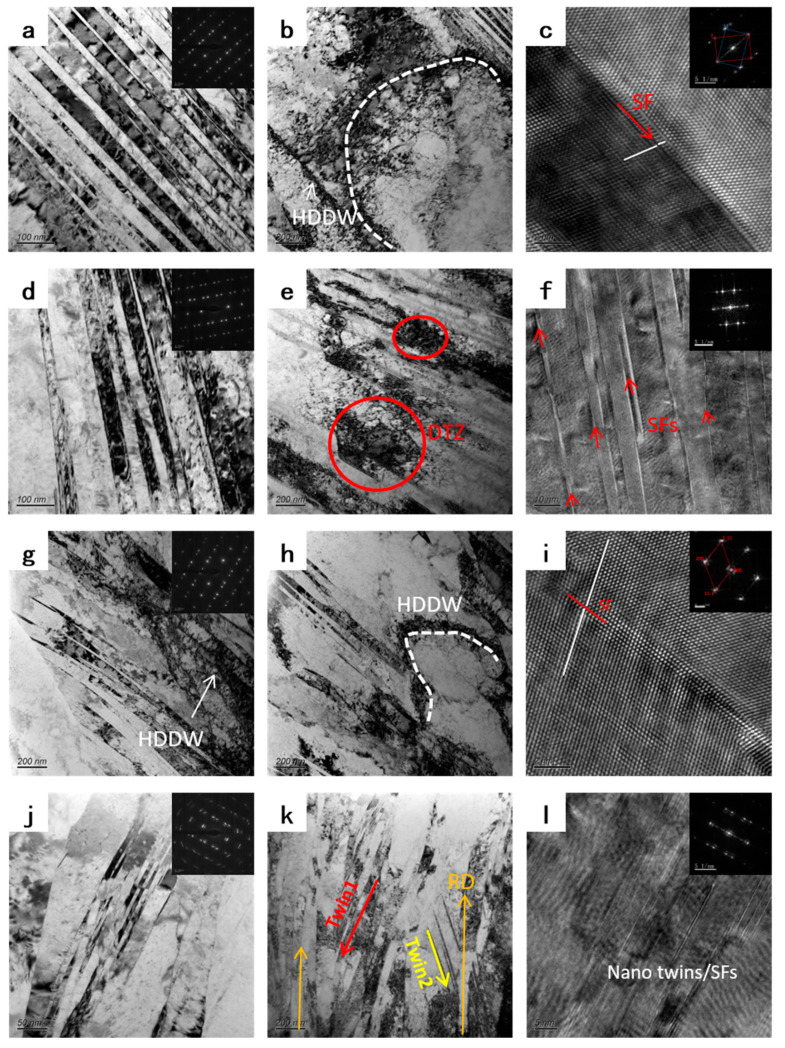
TEM images showing the microstructural evolution of RTR samples with different reductions: (**a**–**c**) RTR30, (**d**–**f**) RTR60, (**g**–**i**) RTR80, and (**j**–**l**) RTR90.

Figure 9 shows the microstructure evolution of Cu-Ti-Cr-Mg alloy during the room temperature rolling process. A large number of deformation twins and high-density dislocation walls (HDDWs) can be observed in the RTR30 sample (Figure 9a,b). Figure 9c is the typical high-resolution TEM image of the twin boundary in Figure 9a, and the stacking fault (SF) is observed along the {111} plane. With the increase of the rolling reduction, the density of nano-twins (twins with a thickness between 10 and 100 nm) in alloys increases, and the average size of nano-twins decreases. The dislocation tangling zones (DTZ) are observed in the samples (Figure 9e). Figure 9f is the high-resolution image of Figure 9d, showing a large number of SFs (indicated by red arrows in the figure) and nano-twins in the sample. In the RTR80 sample, the HDDW interlaces with the twins, and the twins begin to deform and distort. When the rolling reduction increases to 90%, the deformation twins are further distorted, as evidenced by the elongation of the diffraction spot in the illustration of Figure 9j. Figure 9l is the high-resolution image of Figure 9j, and there is a circle of satellite spots around each diffraction spot in the corresponding FFT figure, suggesting that the twin distortion leads to a slight change in orientation. In addition, two new twin systems intersecting the rolling direction appear in the twin crystals parallel to the rolling direction.

**Figure 10:**
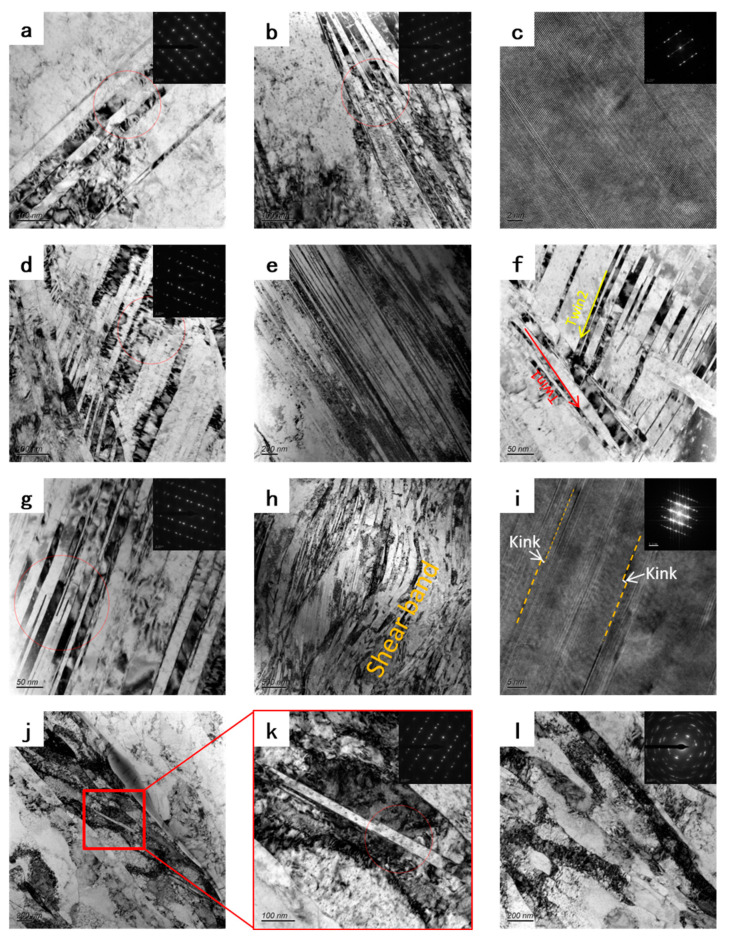
TEM images showing the microstructural evolution of CTR samples with different reductions: (**a**–**c**) CTR30, (**d**–**f**) CTR 60, (**g**–**i**) CTR 80, and (**j**–**l**) CTR 90.

Figure 10 shows the microstructure evolution of Cu-Ti-Cr-Mg alloy during the CTR process. The microstructure evolution in CTR samples is similar to that in RTR samples during rolling, but the evolution process is significantly faster than that in RTR samples. The twin size in the CTR30 sample is smaller than that in the RTR30 sample. Two kinds of deformed twin systems are shown in Figure 10d,f, which can be commonly found in the CTR60 sample. The post-activated twin divides the primary twin into smaller nanostructure. After an 80% reduction at cryogenic temperature, the twins in the sample are distorted and deformed, resulting in a large number of shear bands. In the high-resolution image (Figure 10i), many kinks between twins can be observed. Further increasing the rolling reduction to 90%, the deformation twins fracture, leading to the formation of a large number of cell block tissues in the sample, and only a small number of very small-size twins remain.

## 4. Discussion

### 4.1. Effects of Deformation Temperature on the Texture of the Alloy

**Figure 11:**
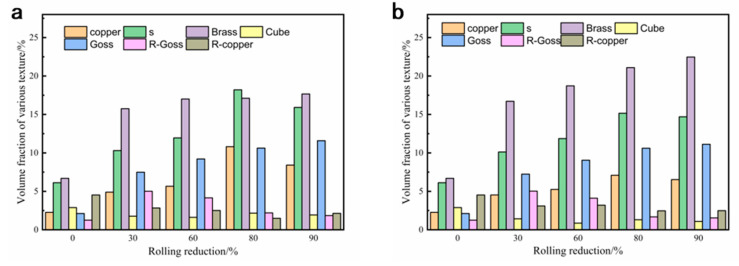
Variation in the volume fraction of various texture components with different rolling reductions at (**a**) room temperature and (**b**) cryogenic temperature.

Rolling textures in face-centered cubic metals usually appear on α lines (<110> parallel to ND) and β lines (<110> tilted 60° in the RD direction) in the orientation space. The main textures on the α lines are Goss texture and Brass texture, while the main textures on the β line are Brass texture, S texture, and Copper texture. The Miller indices of the rolling texture components for FCC alloys are given in Table 4 [26]. The formation of a particular texture is closely related to the properties of the material, such as stacking fault energy (SFE). During the rolling process, the Copper texture is usually found in high SFE materials, while the Brass texture tends to form in low SFE materials [27]. In order to analyze the influence of rolling conditions on the evolution of the texture, the volume fraction of each texture under different rolling conditions was calculated using the Texture Call software. The volume fraction of these components with different rolling reductions is plotted in Figure 11.

As shown in Figure 11, the volume fractions of Brass {011}<211> texture and S {123}<634> texture in the solid-solution treated sample are relatively high, and the other components are relatively weak. During the rolling process, the Brass {011}<211> texture and Goss {011}<100> texture in both of the RTR and CTR samples increase with the increasing reduction. However, the volume fraction of the Brass texture in the CTR samples is consistently higher than that of the RTR samples at any given rolling reduction. The priority development of Brass texture during the CTR process could be attributed to extensive twins or inhibition of cross-slip [28]. As mentioned above, CTR samples have higher twin densities and smaller twin sizes than RTR samples. Besides, the cross-slip process is temperature sensitive and inhibited at cryogenic temperature, which could be the reason for the preferential development of Brass texture. At the beginning of deformation (30% reduction), the volume fraction of R-Goss texture increases, and then its volume fraction decreases with the increase of the rolling reduction. Meanwhile, the Cube {001}<100> texture remains at a low level after further reduction.

It is worth noting that the contents of the Copper texture and the S texture increase first and then decrease with the increase of rolling reduction, and both of them reach the peak values when the rolling reduction is 80%. In the plastic deformation of copper alloys, the Copper {112}<111> texture in the <111> direction has poor plastic deformation ability compared to that in the <100> and <110> directions due to the fewer slip systems and lower Schmid factor [29]. This could be attributed to the relatively low elongation of rolled samples at the rolling reduction of 80% (as shown in Table 2).

### 4.2. Effect of Rolling Temperature on Microstructure and Properties of the Alloy

There is a significant difference in microstructure and mechanical properties of the Cu-Ti-Cr-Mg alloys rolled at room temperature and cryogenic temperature. The yield strength, tensile strength, and work hardening rate of the composites increase with the decrease in temperature. Dislocation slips and deformation twins are two common deformation mechanisms in the plastic deformation of copper alloys. These two mechanisms are in competition during deformation, and the dominance mainly depends on the SFE of the material. Furthermore, the deformation mechanism also depends on the processing parameters, especially the strain rate and the deformation temperature [30]. Normally, the twin boundaries act as strong obstacles to the dislocation motion, leading to high strain-hardening. At the cryogenic temperature, the critical resolved shear stress (CRSS) of dislocation slip increases significantly, leading to the inhibition of dislocation motion, and the CRSS of the deformation twins is virtually unaffected by temperature [31]. Hence, deformation at the cryogenic temperature can promote the occurrence of deformation twins.

It has been reported that metals with relatively low SFEs (about 18~45 mJ/m^2^) are conducive to the formation of deformation twins in FCC alloys [32]. The Cu-Ti alloy has a low SFEs of about 20 mJ/m^2^ [33], and the deformation twins become their typical characteristic. In this study, deformation twins appear in both RTR and CTR samples, as shown in Figure 4 and Figure 6. It indicates that even at room temperature, the SFs in the alloy could transition to twins. Furthermore, the evolution of twins during the CTR process is significantly faster than that of the RTR process, and there are more twins accumulated in the CTR60 sample than that in the RTR60 sample (Figure 6 and Figure 7), which may be related to the decrease in the SEF value of the alloy at a cryogenic temperature [34].

**Figure 12:**
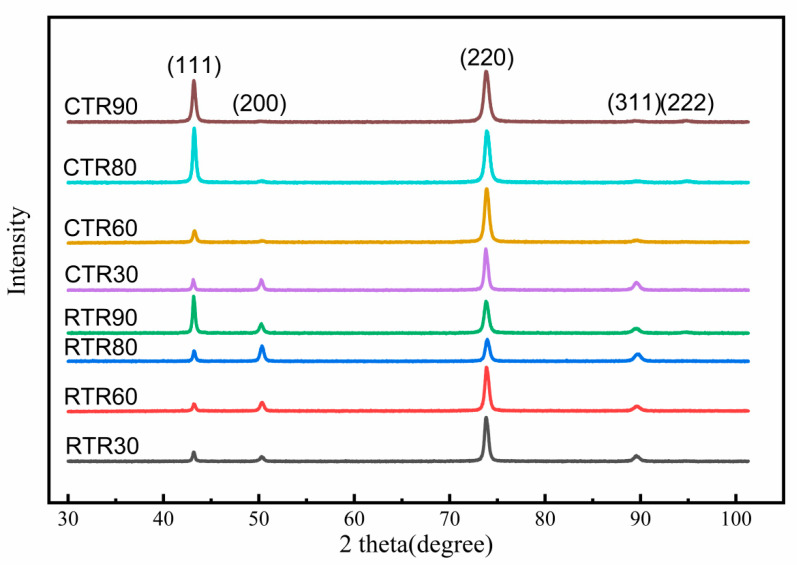
X-ray diffraction results of the RTR samples and CTR samples.

On the other hand, cryogenic temperature inhibits the slip and annihilation of dislocations, preserving a high density of dislocations in the samples and enhancing the mechanical properties of the matrix. Figure 12 shows the X-ray diffraction results of the RTR and CTR samples. The improved Williamson-Hall method [35] was used to calculate the micro-strain of the material according to the width of the XRD diffraction peak. The formula is as follows:

$$\delta_{hkl}\cos\theta_{hkl}=\frac{k\lambda }{d}+2\varepsilon \sin\theta_{hkl}$$

where $\delta_{hkl}$ is the half-height width of the diffraction peak, $\theta_{hkl}$ is the diffraction angle of the (hkl) crystal plane, *k* is a constant (about 0.9) [36], λ is the wavelength of the incident wave (0.154 nm of Cu-$K_{\alpha }$), d is the average grain size, and ε is the slope of the fitted line. The dislocation density can be calculated by [37]:

$$\rho =\frac{16.1\varepsilon^{2}}{b^{2}}$$

where ρ is the dislocation density of the alloy, and *b* is the Burgers vector of the matrix (0.255 nm) [38].

**Figure 13:**
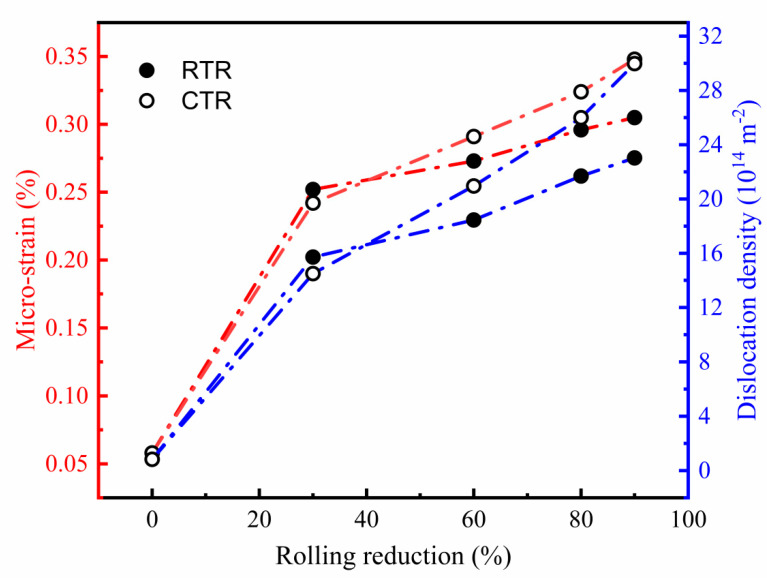
Variation of micro-strain and dislocation density after different rolling reductions.

Figure 13 shows the variation of micro-strain and dislocation density of samples after different rolling reductions. With the increase of rolling reduction, the dislocation density shows an upward trend, however, the rate of increase of dislocation density gradually slows down. Micro-strain has the same trend as the dislocation density with the rolling reduction. It is worth noting that when the rolling reduction is 30%, the deformation energy storage of the RTR sample is not enough to cause dynamic recovery and dynamic recrystallization. Therefore, the dislocations are accumulated, and the hindrance effect of cryogenic temperature on dislocation motion is obvious. When the rolling reduction is higher than 30%, the dislocation density of the CTR samples is higher than that of the RTR samples, and the difference increases with the increase of the rolling reduction. After rolling by 90% reduction, the RTR and CTR samples have dislocation densities of 23.03$\;\times \;10^{14}{\;m}^{-2}$ and 29.98$\;\times \;10^{14}{\;m}^{-2}$, respectively. With the increase of rolling reduction, the dynamic recovery in the RTR samples continuously increases, and the decrease in dislocation density originates from the recovery increases. However, the inhibition of dynamic recovery by cryogenic temperature allows more dislocations to be retained in CTR samples, resulting in a higher dislocation density.

According to the above results and discussion, it can be safely suspected that the cryogenic temperature significantly accelerates the evolution of microstructure during rolling, resulting in much more nano-twins and dislocations and better mechanical properties of the CTR alloy (as shown in Table 2).

## 5. Conclusions

1. The Cu-Ti-Cr-Mg alloy has a higher work hardening rate at cryogenic temperature. The yield strength and tensile strength of the CTR90 sample are 723 MPa and 796 MPa, increasing by about 25% and 23% compared with those of the RTR90 sample, respectively.
2. The solid solution-treated Cu-Ti-Cr-Mg alloy has weak Brass texture and S texture, and the textures change to Brass and Goss textures during the deformation. The cross-slip is inhibited at cryogenic temperature, leading to the priority development of Brass texture in cryogenic rolling. The microstructure evolution during the rolling deformation is related to dislocation slip and nano-twins deformation, and the cryogenic temperature promotes the deformation progress.
3. With the increase of rolling reduction, the dislocation density of Cu-Ti-Cr-Mg alloy increases significantly during CTR and RTR processes, finally reaching 29.98$\;\times \;10^{14}{\;m}^{-2}$ and 23.03$\;\times \;10^{14}{\;m}^{-2}$, respectively. The difference in the dislocation densities between the CTR and RTR samples increases with the increasing rolling reduction. The enhanced mechanical properties of the alloy after cryogenic rolling are due to cryogenic temperature inhibiting the dynamic recovery and dynamic recrystallization during rolling.

## Figures and Tables

**Table 1:** The nominal and measured compositions of the alloy.

Composition Element	Ti	Cr	Mg	Cu
Nominal composition(wt.%)	1.8	0.4	0.1	Bal.
Measured composition(wt.%)	1.85	0.39	0.097	Bal.

**Table 2:** The details of the abbreviation.

Rolling Reduction	Room Temperature	Cryogenic Temperature
30%	RTR30	CTR30
60%	RTR60	CTR60
80%	RTR80	CTR80
90%	RTR90	CTR90

**Table 3:** Mechanical properties of the Cu-Ti-Cr-Mg alloy after different rolling treatments.

	ST	RTR30	RTR60	RTR80	RTR90	CTR30	CTR60	CTR80	CTR90
Yield strength (MPa)	262 ± 12	411 ± 1	506 ± 2	560 ± 11	588 ± 13	474 ± 10	568 ± 13	676 ± 10	723 ± 6
Tensile strength (MPa)	319 ± 30	442 ± 2	552 ± 7	605 ± 2	634 ± 3	513 ± 1	627 ± 11	702 ± 14	796 ± 3
Elongation (%)	46.8 ± 0.3	21.6 ± 1.0	16.3 ± 0.3	14.6 ± 0.1	15.6 ± 0.5	20.3 ± 0.6	16.2 ± 1.8	14.6 ± 0.4	16.0 ± 0.2

**Table 4:** Important rolling texture components for FCC alloy [26].

Texture Component	Miller Indices	Euler Angles (°)		
		$\varphi_{1}$	*Φ*	$\varphi_{2}$
Cube	{001}<100>	0	0	0
Copper	{112}<111>	90	35	45
Brass	{011}<211>	55	90	45
Goss	{011}<100>	0	45	0
S	{123}<634>	59	37	63
R-Goss	{011}<011>	0	90	45
R-Copper	{112}<011>	0	35	45

## Data Availability

The data used to support the findings of this study are included within the article.
